# Antenatal depression and its predictors among HIV positive women in Sub-Saharan Africa; a systematic review and meta-analysis

**DOI:** 10.3389/fpsyt.2024.1385323

**Published:** 2024-06-11

**Authors:** Gossa Fetene Abebe, Melsew Setegn Alie, Amanuel Adugna, Daniel Asemelash, Tamirat Tesfaye, Desalegn Girma, Abyot Asres

**Affiliations:** ^1^ Department of Midwifery, College of Medicine and Health Sciences, Mizan-Tepi University, Mizan, Ethiopia; ^2^ School of Public Health, College of Medicine and Health Sciences, Mizan-Tepi University, Mizan Aman, Ethiopia; ^3^ Department of Laboratory, College of Medicine and Health Sciences, Mizan Tepi University, Mizan Aman, Ethiopia; ^4^ College of Medicine and Health Sciences, Hawassa University, Hawassa, Ethiopia

**Keywords:** antenatal depression, HIV-positive women, Sub-Saharan Africa, systematic review, meta-analysis

## Abstract

**Background:**

Antenatal depression in Human Immunodeficiency Virus (HIV) positive pregnant women can have significant adverse effects on both the mother and newborns, yet it is often overlooked in pregnancy care in Sub-Saharan Africa (SSA). Despite this, there is limited data on the combined prevalence of antenatal depression and its predictors among HIV-positive women in the region.

**Objective:**

To assess the pooled prevalence of antenatal depression and its associated factors among HIV-positive women in SSA.

**Methods:**

All primary cross-sectional studies published before 1^st^ January/2024, were included. We conducted searches in relevant databases; PubMed, HINARI, Web of Science, PsycINFO, Psychiatry Online, ScienceDirect, and Google Scholar. The Joanna Briggs Institute checklist was used to critically appraise the selected studies. To assess heterogeneity among the studies, we utilized the I^2^ test. Publication bias was evaluated using a funnel plot and Egger’s test. The forest plot was used to present the combined proportion of antenatal depression and odds ratio, along with a 95% confidence interval.

**Results:**

The pooled prevalence of antenatal depression among HIV-positive women in Sub-Saharan Africa was found to be 30.6% (95% CI, 19.8%-41.3%). Factors significantly associated with antenatal depression among HIV-positive women in SSA included being unmarried (AOR: 3.09, 95% CI: 1.57 – 6.07), having a previous history of depression (AOR: 2.97, 95% CI: 1.79 – 4.91), experiencing intimate partner violence (IPV) (AOR: 2.11, 95% CI: 1.44 – 3.09), and experiencing stigma (AOR: 1.36, 95% CI: 1.05 – 1.76).

**Conclusion:**

High prevalence of antenatal depression among HIV-positive women in SSA underscores the need for prioritizing identification and management. Interventions addressing factors like IPV and stigma, along with training for healthcare providers in recognizing symptoms and providing support, are recommended.

**Systematic Review Registration:**

https://www.crd.york.ac.uk/PROSPERO/, identifier CRD42024508236.

## Introduction

Antenatal depression is a common mental health disorder that affects pregnant women worldwide (1), with lower and middle-income countries bearing the highest burden (2). In Sub-Saharan Africa (SSA), where the prevalence of HIV is high (3), antenatal depression is a significant public health concern among HIV positive women (4–6). Serious forms of depression characterized by the idea of suicide or self-harming are common during pregnancy (7).

Antenatal depression in HIV-positive pregnant women can accelerate HIV disease progression (8, 9) and raise maternal morbidity and mortality rates (8, 10). Additionally, HIV-positive women with depression tend to have poor adherence to antiretroviral therapy (ART) (9, 11–13). Untreated depression during pregnancy is also a risk factor for postpartum depression (14), and reduces the likelihood of exclusive breastfeeding for newborns (15). Furthermore, antenatal depression has a lasting impact on both mothers, infants, and children across generations (16–20).

By 2030, depression and HIV/AIDS are expected to be the main global disease burdens (21). To improve mental health, national and international strategies have been implemented for vulnerable groups like postpartum women, and those living with HIV/AIDS (22, 23). However, in SSA, antenatal depression with HIV-positive women is prevalent, as the primary studies conducted in different places in the region have indicated (2, 13, 24–34). HIV has been documented to have effects on the fetus, including low birth weight, preterm births, small for gestational age, delayed cognitive and language development, behavioral problems, poor academic performance, and emotional issues (35–37).

Previous studies in SSA countries have reported varying rates of antenatal depression among HIV-positive pregnant women, ranging from 6.5% to 67% (27, 32), and being unmarried, unemployed, unplanned pregnancy, length of time on ART, experienced intimate partner violence during the current pregnancy, ART non-adherence, experienced internalized AIDS stigma, poor social support, and history of depression have been found to be associated with developing antenatal depression among HIV-positive pregnant women (2, 4, 13, 24–34, 38–40).

These primary studies were conducted in a fragmented manner across different SSA countries, and the pooled prevalence of antenatal depression among HIV-positive pregnant women has not been determined in SSA. Therefore, in this systematic review and meta-analysis, we aimed to synthesize the available evidence on the pooled prevalence of antenatal depression and its predictors among HIV positive women in SSA. The findings will provide valuable insights into the burden of antenatal depression in this population, inform the development of effective interventions, and contribute to the achievement of Sustainable Development Goal (SDG) 3, which aims to ensure healthy lives and promote well-being for all at all ages by 2030.

## Methods

### Study setting and period

We included studies conducted among countries listed in the Sub-Saharan Africa region. The search period was from October 1, 2023 to January 1, 2024.

### Search strategies

This systematic review has adhered to the Preferred Reporting Item for Systematic Review and Meta-Analyses (PRISMA) guideline and checklist (41). PubMed, HINARI, Web of Science, PsycINFO, Psychiatry Online, ScienceDirect, and Google Scholar were searched for relevant studies on antenatal depression among HIV positive women in Sub-Saharan Africa. Moreover, reference lists of eligible studies were retrieved to account for the missed studies in the database searching. All studies reporting the proportion or prevalence of antenatal depression among HIV positive women were the target of this review. All primary cross-sectional studies published before January 1, 2024 were included. The search was done using keywords such as antenatal depression, or depression in antenatal period, depression during pregnancy and associated factors, or determinants, or predictors, and country found in Sub-Saharan Africa. Combinations of Boolean operators (AND, OR), free keywords, and MeSH terms were used in the search process (**Additional File 1**).

### Eligibility criteria

The inclusion criteria encompassed: (1) studies conducted in Sub-Saharan Africa country, (2) all cross-sectional studies, (3) studies reporting the prevalence of antenatal depression and/or associated or determinant factors or predictors, and (4) studies published as full-length articles in English. Whereas, conference papers or abstracts, articles lacking full texts, anonymous reports, editorial reports, and qualitative studies were not included in this study.

### Data extraction

The data from all the studies included in the analysis were collected and organized in a customized Microsoft Excel spreadsheet. This extraction process was carried out by three authors (GA, DG, AA, and MA) using a standardized extraction form. In case of any discrepancies between the data extractors, discussions were held and resolved by the three authors (AA, TT, and DA). We extracted important information from each study, which included the author’s name, publication year, antenatal depression event, prevalence, country, study design, and associated factors such as odds ratios.

### Quality assessment/critical appraisal

The Joanna Briggs Institute (JBI) Critical Appraisal Checklist for cross-sectional studies was used to assess the quality of the study (42). Two authors (GA and AA) independently assessed the quality of each article using a critical appraisal checklist adapted from JBI. During the critical appraisal process, if any discrepancies arose, the reviewers held discussions led by the fourth author (TT) to address and resolve the issues. Finally, a total of 13 studies that fulfill the inclusion criteria were considered in this meta-analysis (**Additional File 2**).

### Statistical analysis

Data entry was performed using the Microsoft Excel Database, and the entered data was subsequently imported into R software version 4.1.3 for further analysis utilizing the Meta-package. The heterogeneity between studies was evaluated using the I^2^ index (43), where values of 25%, 50%, and 75% indicated low, medium, and high heterogeneity, respectively (44). Publication bias was evaluated by visually inspecting the symmetry of the funnel plot and conducting the Egger test. A p-value of less than 0.05 on the Egger test indicated the presence of publication bias among the included studies. In order to identify potential sources of heterogeneity, a univariate Meta-regression analysis was conducted, taking into account the sample size and year of publication. Additionally, a leave-one-out sensitivity analysis was performed, systematically removing one study at a time to assess the impact of each individual study on the overall estimate (45). Furthermore, a trim-and-fill analysis was conducted to assess the extent of distortion in the pooled prevalence of male involvement caused by publication bias. The results are presented in a forest plot, displaying a point estimate along with 95% confidence intervals. All analyses were performed using R software version 4.1.3.

## Result

### Characteristics of the studies included in the meta-analysis and systematic review

**Figure 1:**
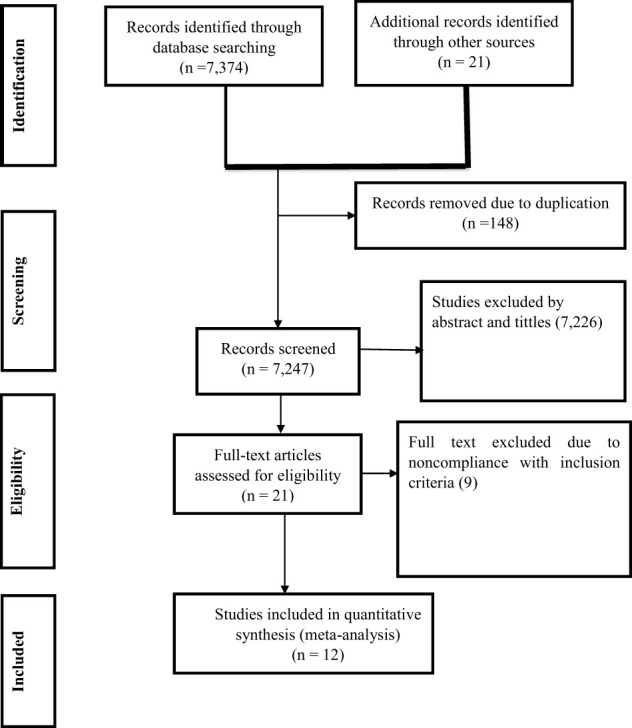
PRISMA flow chart showing the process of search and selection of studies included in the systematic review and meta-analysis.

Out of the 7,395 articles identified through different search strategies, 148 records were eliminated due to duplication. An additional 7,226 articles were excluded because their titles and abstracts were deemed inappropriate. Additionally, 9 were excluded due to noncompliance with the inclusion criteria. Finally, only 12 studies met the eligibility criteria and were considered in this study (**Figure 1**). This study incorporated a total of 7,109 participants from 12 studies, with sample sizes ranging from 198 to 2298 (13, 24–33, 38) (**Table 1**).

**Table 1:** Characteristics of studies included in the meta-analysis.

Authors	Country	Study design	N	Number of women diagnosed for antenatal depression	Prevalence
Abate H. et al., 2021 (24)	Ethiopia	Cross-sectional study	291	84	28.7
Abebe W. et al., 2022 (25)	Ethiopia	Cross-sectional study	368	175	46.7
Harrington, B. et al., 2019 (26)	Malawi	Cross-sectional study	725	69	9.5
Jones M. et al., 2021 (27)	African cohort study	Cross-sectional study	214	14	6.5
Osborn, L. et al., 2022 (30)	Kenya	Cross-sectional study	824	71	9
Regan, M. et al., 2023 (32)	Tanzania	Cross-sectional study	2298	1540	67
Schwartz, S. et al., 2023 (38)	Ethiopia	Cross-sectional study	423	159	37.6
Shoptaw, S. et al., 2018 (33)	Malawi	Cross-sectional study	299	38	13
Desalegn et al., 2022 (13)	Ethiopia	Cross-sectional study	606	215	36.4
Nyamukoho et al., 2019 (29)	Zimbabwe	Cross-sectional study	198	78	39.4
Peltzer, et al., 2016 (31)	South Africa	Cross-sectional study	663	323	48.7
Ngocho, et al., 2019 (28)	Tanzania	Cross-sectional study	200	50	25

Key; N = Total sample size.

### Pooled prevalence of antenatal depression among HIV positive women in SSA

**Figure 2:**
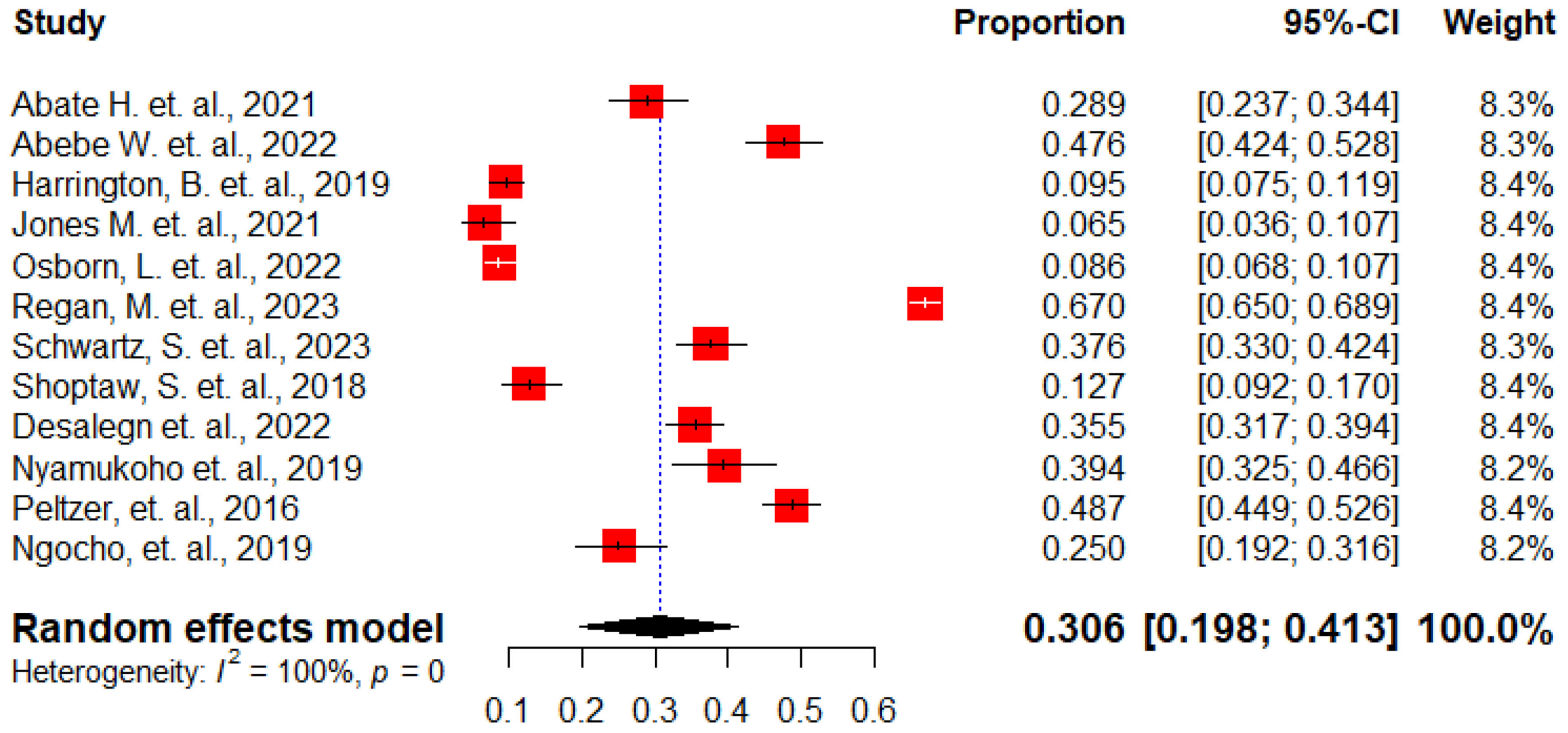
The forest plots show the pooled prevalence of antenatal depression among HIV positive women in Sub-Saharan Africa, 2024.

**Figure 3:**
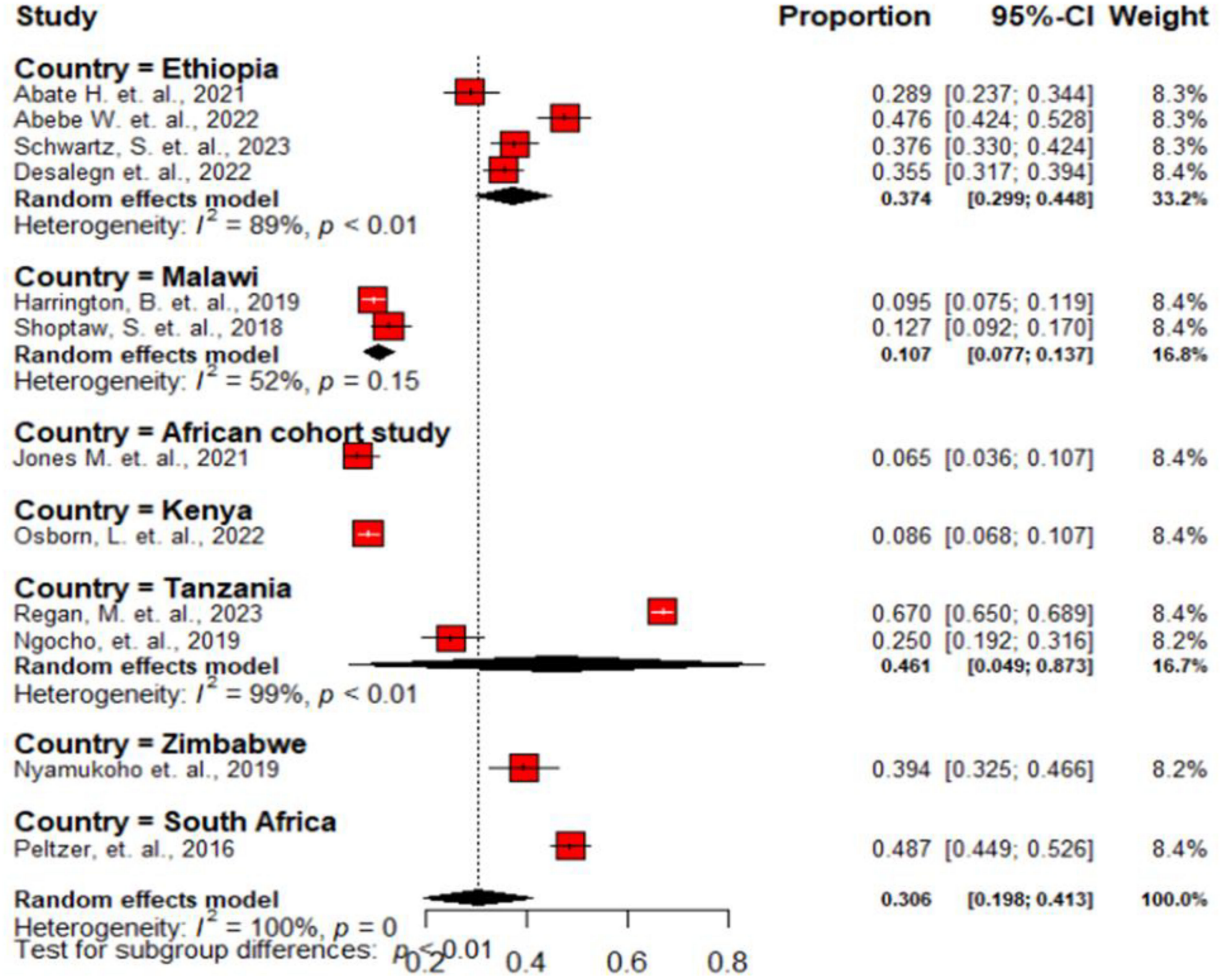
Forest plot showing the subgroup analysis of the pooled prevalence of antenatal depression among HIV positive women in Sub-Saharan Africa, 2024.

The overall pooled prevalence of antenatal depression among HIV positive women in SSA was 30.6% (95% CI, 19.8%-41.3%) (**Figure 2**). A subgroup analysis was conducted by country in SSA, revealing that the lowest prevalence was reported from African cohort, 6.5%, while the highest prevalence was reported from South Africa, 48.7% (**Figure 3**).

### Assessment of publication bias

**Figure 4:**
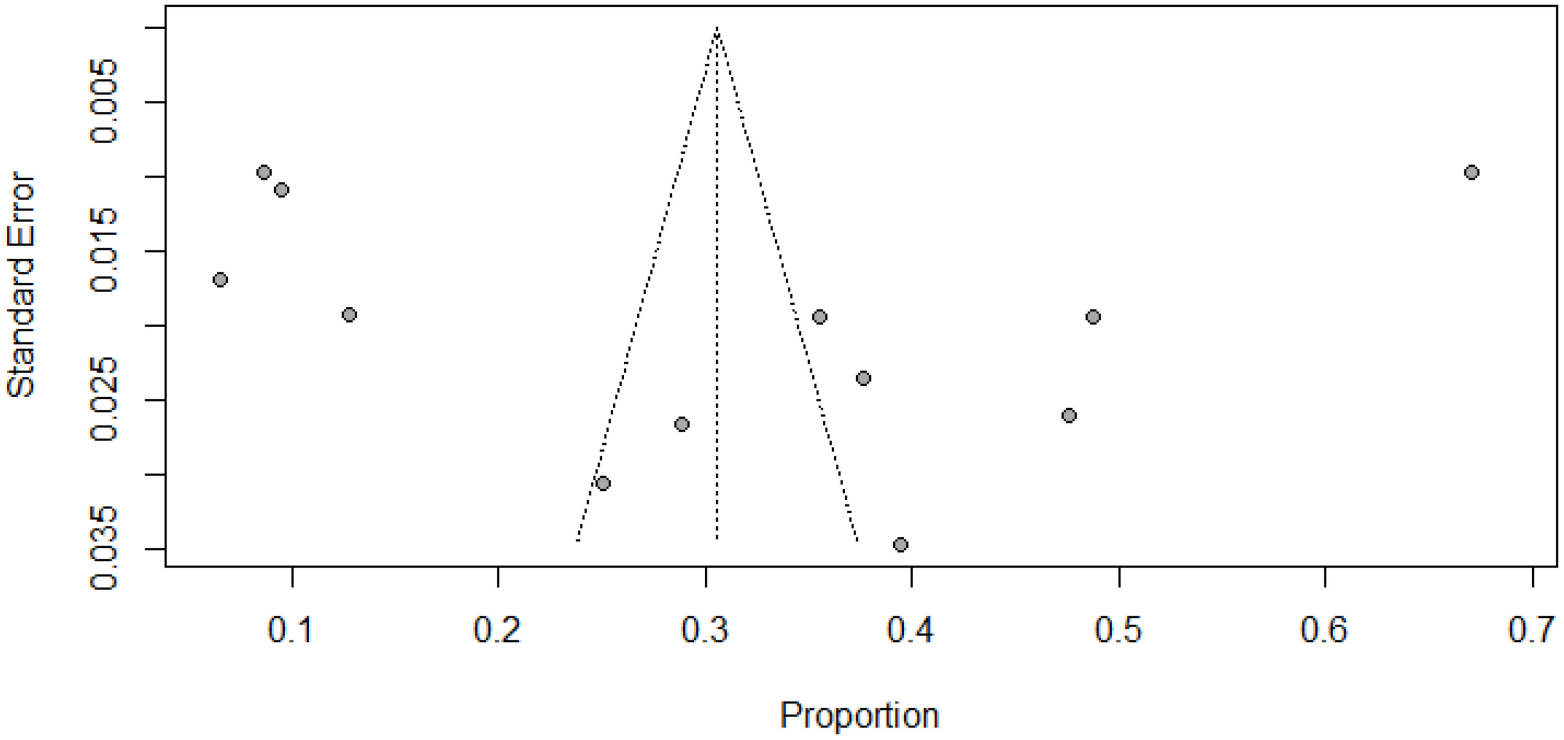
Funnel plot showing publication bias among studies used to compute the pooled prevalence of antenatal depression among HIV positive women in SSA, 2024.

We used both subjective and objective measures to evaluate publication bias. First, funnel plot visual inspection was done. Then, we employed the Egger’s test. Our findings revealed that the funnel plot displayed symmetrical distribution of studies in the line of effect (**Figure 4**). The Egger’s test also noted a non-significant value for publication bias (*P* = 0.916).

### Meta-regression and sensitivity analysis

The impact of study characteristics on the pooled estimate was assessed using meta-regression. However, none of the study characteristics, such as publication year and sample size, showed any association with the pooled estimates (P > 0.05) (**Table 2**).

**Table 2:** Meta regression analysis of factors affecting between study heterogeneity.

Variables	Coefficients	P-value
Publication years	0.027 (-0.036, 0.0485)	0.547
Sample size	0.0001 (-0.0001, 0.0003)	0.097

**Figure 5:**
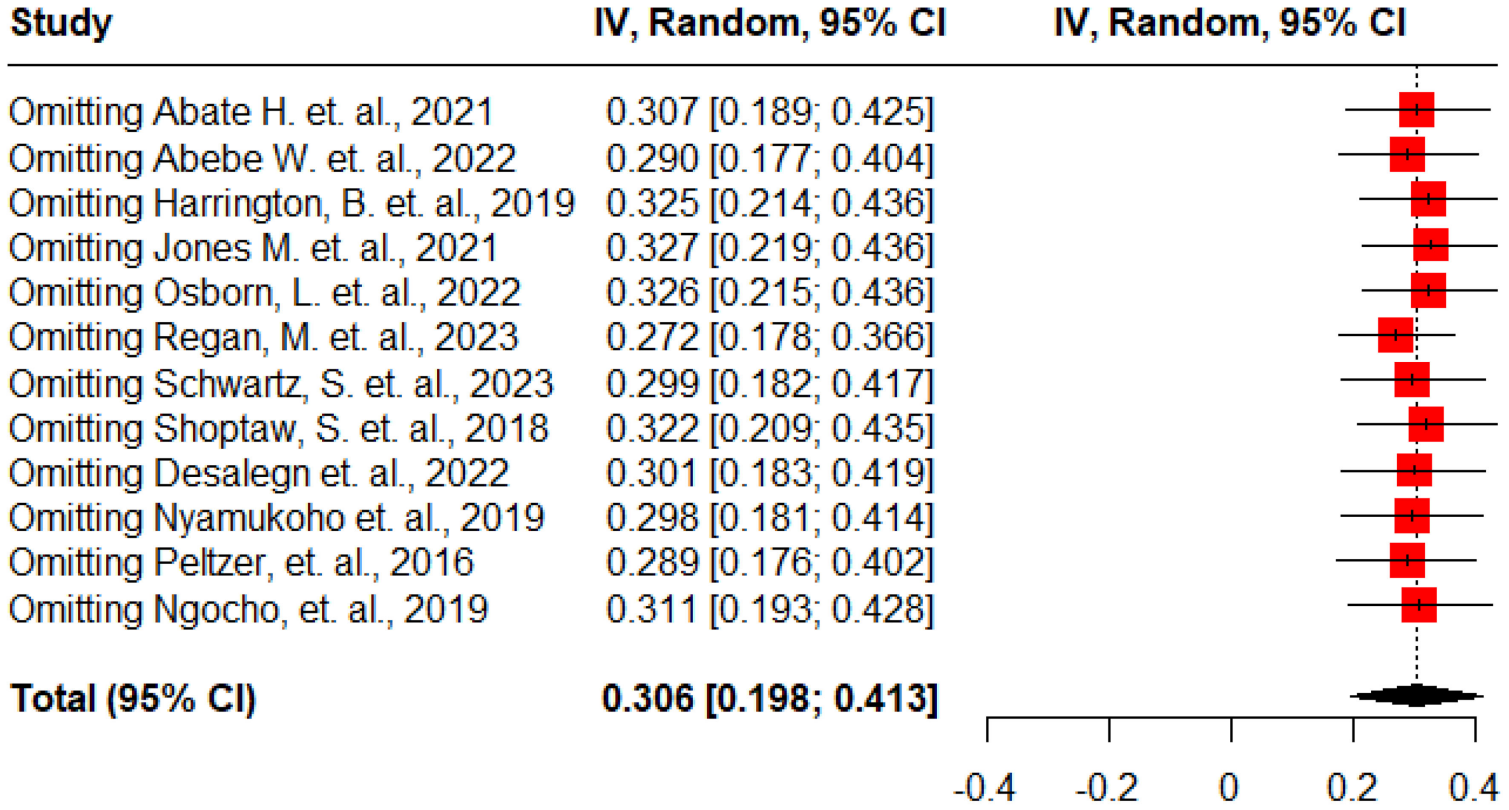
Sensitivity analysis for the pooled prevalence of antenatal depression among HIV positive women in Sub-Saharan Africa, 2024.

To assess the impact of each study on the pooled estimates, a thorough sensitivity analysis was conducted by systematically excluding one study at a time. The results showed that almost all studies made comparable contributions to the overall prevalence of antenatal depression among HIV-positive women in SSA. When each study was omitted from the analysis, the pooled prevalence of antenatal depression ranged from 27.2% to 32.7% (**Figure 5**).

### Factors associated with antenatal depression among HIV positive women in SSA

In this meta-analysis, we found significant factors associated with antenatal depression among HIV-positive women in SSA. These factors include being unmarried, employed, a previous history of depression, experiencing IPV, experiencing stigma, and ART non-adherence.

**Figure 6:**
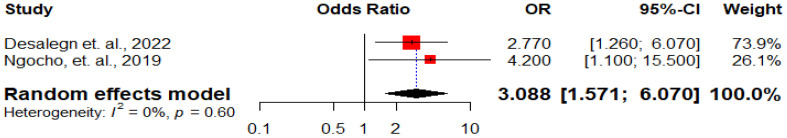
Forest plot displaying the association between being unmarried and antenatal depression among HIV positive women in Sub-Saharan Africa, 2024.

Two studies (13, 28), involving a total of 806 participants, examined the association between unmarried HIV-positive pregnant women and developing antenatal depression. The random effect model analysis revealed a significant association between being unmarried and developing of antenatal depression. Thus, the odds of developing antenatal depression were three times higher among unmarried HIV-positive pregnant women compared to those married women (AOR: 3.09, 95% CI: 1.57 – 6.07) (**Figure 6**).

**Figure 7:**
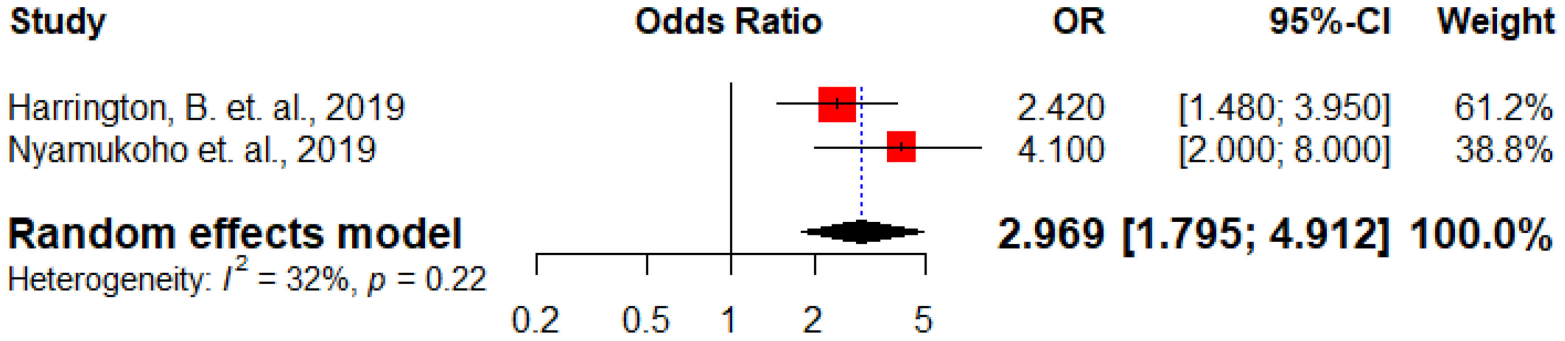
Forest plot displaying the association between previous history of depression and antenatal depression among HIV positive women in Sub-Saharan Africa, 202.

Two studies (26, 29) examined the correlation between a history of depression and the occurrence of antenatal depression. The overall estimate indicated that the odds of occurrence of antenatal depression were three times higher among HIV positive women with a previous history of depression compared to their counterparts (AOR: 2.97, 95% CI: 1.79 – 4.91) (**Figure 7**).

**Figure 8:**
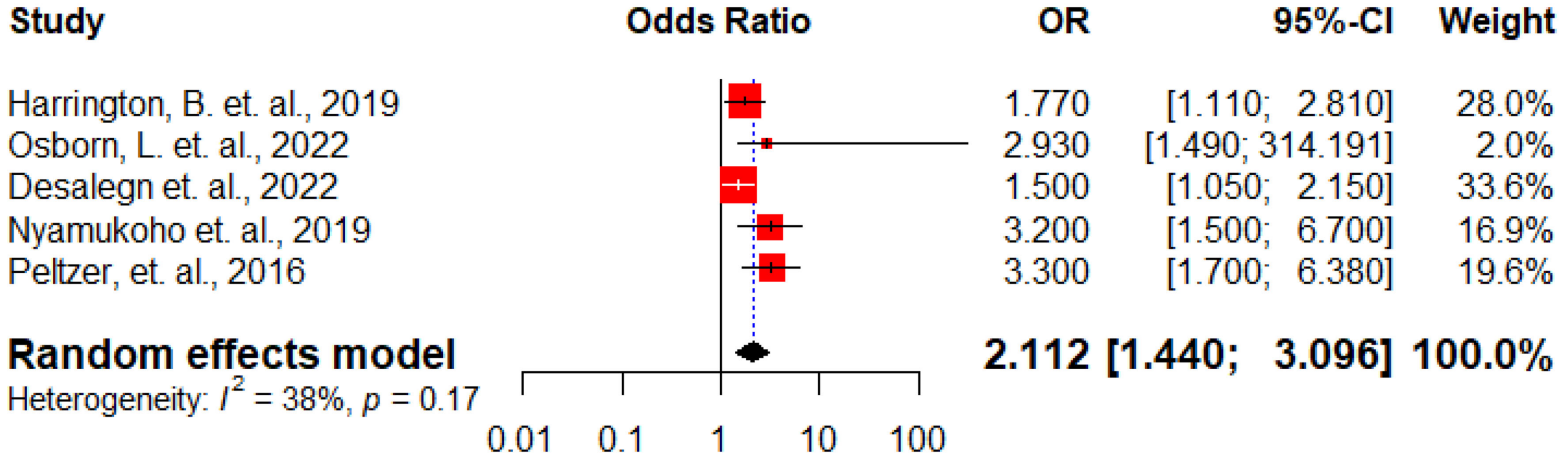
Forest plot displaying the association between experiencing IPV and antenatal depression among HIV positive women in Sub-Saharan Africa, 2024.

Five studies (13, 26, 29–31) demonstrated the correlation between HIV-positive pregnant women experiencing intimate partner violence (IPV) and the occurrence of antenatal depression. The overall analysis indicated that HIV-positive pregnant women who experienced IPV had twice the odds of developing antenatal depression compared to their counterparts (AOR: 2.11, 95% CI: 1.44 – 3.09) (**Figure 8**).

**Figure 9:**
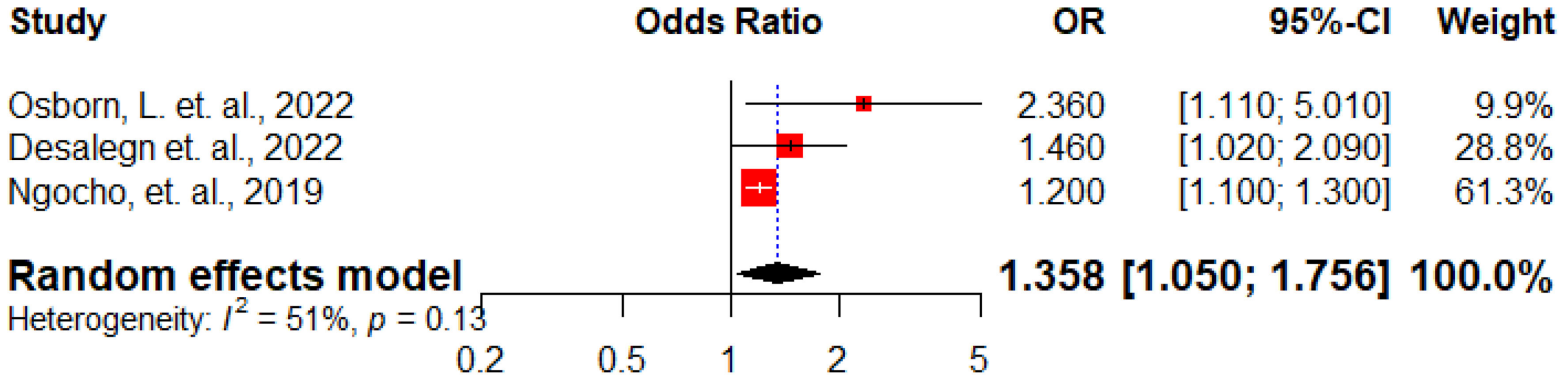
Forest plot displaying the association between presence of stigma and antenatal depression among HIV positive women in Sub-Saharan Africa, 2024.

Three studies (13, 28, 30) assessed the correlation between stigma experienced by HIV-positive pregnant women and the occurrence of antenatal depression. The overall analysis showed that those who had experienced stigma had 1.4 times higher odds of developing antenatal depression compared to those who had not experienced stigma (AOR: 1.36, 95% CI: 1.05 – 1.76) (**Figure 9**).

**Figure 10:**
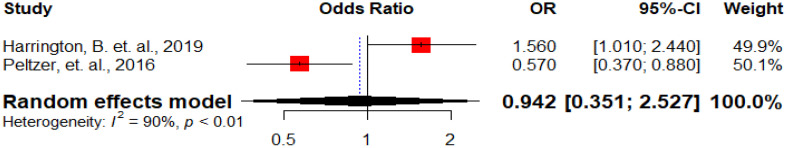
Forest plot displaying the association between being employed and antenatal depression among HIV positive women in Sub-Saharan Africa, 2024.

Inconsistent findings were reported regarding the correlation between employment status and the occurrence of antenatal depression. Harrington et al. (26) found that employed women had higher odds of experiencing antenatal depression, while Peltzer et al. (31) showed a negative association between employment and antenatal depression. The overall analysis indicated no significant association between employment status and antenatal depression (AOR: 0.94, 95%CI: 0.35 – 2.53) (**Figure 10**).

**Figure 11:**
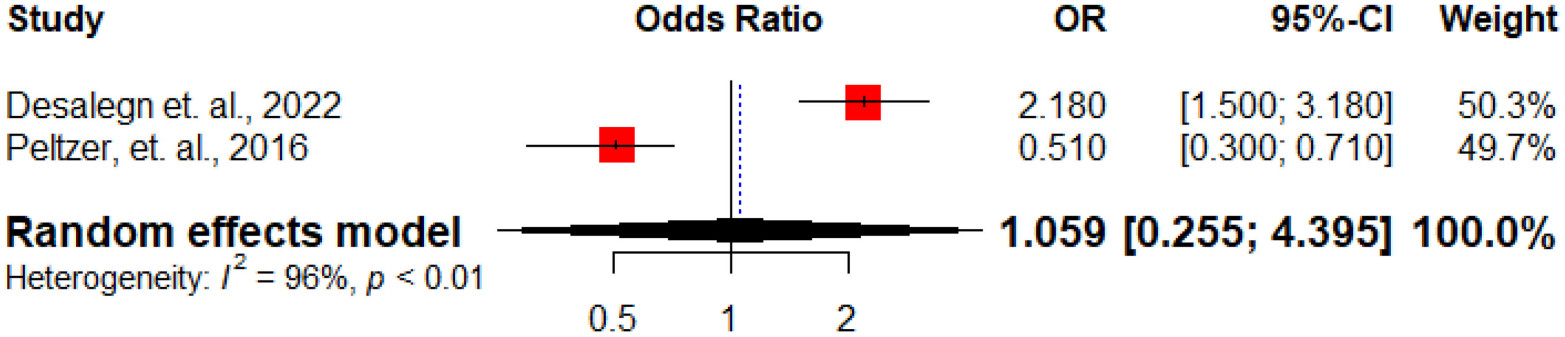
Forest plot displaying the association between ART non-adherence and antenatal depression among HIV positive women in Sub-Saharan Africa, 2024.

Inconsistent findings have been reported regarding the correlation between non-adherence to ART and the occurrence of antenatal depression. Desalegn et al. (13) noted that HIV-positive pregnant women who were not adherent to ART had higher odds of developing antenatal depression, while Peltzer et al. (31) indicated that those who were not adherent to ART had lower odds of developing antenatal depression. The overall analysis showed no significant association between non-adherence to ART and the occurrence of antenatal depression (AOR: 1.06, 95%CI: 0.25 – 4.39) (**Figure 11**).

## Discussion

Despite the significant negative impact of antenatal depression on the health of both women and fetuses in Sub-Saharan Africa, it has received less attention than postnatal depression (46). As a result, there is limited data on the pooled prevalence of antenatal depression and its associated factors among HIV-positive women in the region. Therefore, this systematic review and meta-analysis aim to synthesize the available evidence on the prevalence of antenatal depression and its predictors among HIV-positive women in SSA.

The pooled prevalence of antenatal depression among HIV-positive women in SSA was found to be 30.6% (95% CI, 19.8%-41.3%), consistent with studies done in Ukraine (27%) (47), China (28.5%) (48), and America (36%) (49). However, this finding is higher than a study done in Bangladesh (18%) (50). This divergence might be due to the difference in the gaps in the study period, and source populations; in which most of the participants in the later study (50) included all pregnant women, regardless of HIV status.

On the other hand, this figure is lower than the 44% reported in a study conducted in the United States (51). The difference might be attributed to the timing of the study; for instance, a study in the United States was done during the COVID-19 pandemic and due to its higher transmission rates, HIV-positive pregnant women faced higher rate of depression (52).

According to this meta-analysis, unmarried HIV-positive pregnant women were found to have three times higher odds of developing antenatal depression compared to married women. This could be attributed to the increased vulnerability of single women to economic, social, and psychological hardships, which may contribute to higher rates of depression (53). Research has also shown that partner support plays a significant role in reducing depression during pregnancy (54). Furthermore, societal norms and cultural factors may contribute to the triggering of depression, as single motherhood is often not socially accepted in most African countries.

In line with study done by Biaggi A. et. al., 2016 (55), the odds of the occurrence of antenatal depression among HIV-positive women with a previous history of depression were three times higher compared to their counterparts. This might be due to the physical and hormonal changes occurring during pregnancy and the recurrence of depressive symptoms (56). However, contrary to this finding, a study conducted in Pakistan found that a personal history of previous psychiatric illness was not significantly associated with antenatal depression (57).

Consistent with study done in United States (58), the odds of developing antenatal depression among HIV-positive pregnant women were higher among women experiencing HIV-related stigma as compared to those who had not experienced stigma. This could be attributed to the fact that acquiring HIV, a chronic lifelong disease, often leads to significant levels of stigma. Also, some patients may choose to withdraw from society to avoid this stigma, which can further contribute to feelings of exclusion or loneliness and exacerbate depression.

The study indicated that the odds of developing antenatal depression among HIV-positive women were two times higher among women who experienced IPV as compared to women who doesn’t experience IPV. This could be explained by the fact that psychological, physical, and sexual violence perpetrated by an intimate partner can exacerbate depression (59, 60).

Additionally, the findings of this systematic review indicate that there is no substantial correlation between non-compliance with antiretroviral therapy (ART) and the incidence of antenatal depression.

## Strength and limitation

This systematic review has several strengths. Firstly, we utilized a robust search algorithm to include studies from multiple databases. Secondly, our study provides the first-ever prevalence estimates of antenatal depression among HIV-positive women in Sub-Saharan Africa. Additionally, we identified the factors associated with the development of depression in HIV-positive women, which is crucial for preventive public health efforts. However, there are certain limitations to this review. Firstly, we only included articles published in English. Secondly, the cross-sectional design of the included studies may limit our ability to establish a causal association between antenatal depression in HIV-positive women and the associated factors. Lastly, some factors were only reported in a single study, preventing the calculation of a pooled effect size.

## Conclusion

The high prevalence of antenatal depression among HIV-positive women in SSA and the significant factors associated with it underscore the need for healthcare providers to prioritize the identification and management of antenatal depression in this population. Interventions addressing the underlying factors contributing to antenatal depression should be developed and implemented. Healthcare providers should also be trained to recognize the signs and symptoms of antenatal depression and provide appropriate support and referrals for mental health services when needed. Addressing antenatal depression among HIV-positive women in Sub-Saharan Africa can improve maternal and child health outcomes and reduce the burden of mental illness in this population.

## Data availability statement

The original contributions presented in the study are included in the article/**Supplementary Material**. Further inquiries can be directed to the corresponding author.

## Author contributions

GA: Writing – review & editing, Writing – original draft, Visualization, Validation, Supervision, Software, Resources, Project administration, Methodology, Investigation, Formal analysis, Data curation, Conceptualization. MA: Writing – review & editing, Writing – original draft, Visualization, Software, Methodology, Formal analysis, Data curation, Conceptualization. AAd: Writing – review & editing, Writing – original draft, Visualization, Validation, Software, Methodology, Investigation, Formal analysis, Data curation, Conceptualization. DA: Writing – review & editing, Writing – original draft, Visualization, Validation, Supervision, Software, Data curation, Conceptualization. TT: Writing – review & editing, Writing – original draft, Visualization, Supervision, Software, Formal analysis, Data curation, Conceptualization. DG: Writing – review & editing, Writing – original draft, Visualization, Validation, Software, Methodology, Data curation, Conceptualization. AAs: Writing – review & editing, Writing – original draft, Validation, Software, Methodology, Investigation, Formal analysis.

## References

[1] Austin M Highest N The Expert Working Group . Mental health care in th e perinatal period: Australian clinical practice guideline Centre of perinatal excellenc e depression during pregnancy. (2017).

[2] Dadi AF Miller ER Bisetegn TA Mwanri L . Global burden of antenatal depression and its association with adverse birth outcomes: an umbrella review. BMC Public Health. (2020) 20:1–16. doi: 10.1186/s12889-020-8293-9 32019560 PMC7001252

[3] WHO . HIV and AIDS (2023). Available online at: https://www.who.int/news-room/fact-sheets/detail/hiv-aids.

[4] Chibanda D Benjamin L Weiss HA Abas M . Mental, neurological, and substance use disorders in people living with HIV/AIDS in low-and middle-income countries. JAIDS J Acquired Immune Deficiency Syndr. (2014) 67:S54–67. doi: 10.1097/QAI.0000000000000258 25117961

[5] Arseniou S Arvaniti A Samakouri M . HIV infection and depression. Psychiatry Clin Neurosci. (2014) 68:96–109. doi: 10.1111/pcn.12097 24552630

[6] Kapetanovic S Dass-Brailsford P Nora D Talisman N . Mental health of HIV-seropositive women during pregnancy and postpartum period: a comprehensive literature review. AIDS Behav. (2014) 18:1152–73. doi: 10.1007/s10461-014-0728-9 PMC4120872 24584458

[7] Arachchi NSM Ganegama R Husna AWF Chandima DL Hettigama N Premadasa J . Suicidal ideation and intentional self-harm in pregnancy as a neglected agenda in maternal health; an experience from rural Sri Lanka. Reprod Health. (2019) 16:1–7. doi: 10.1186/s12978-019-0823-5 31729997 PMC6858764

[8] Antelman G Kaaya S Wei R Mbwambo J Msamanga GI Fawzi WW . Depressive symptoms increase risk of HIV disease progression and mortality among women in Tanzania. J acquired Immune deficiency syndr. (2007) 44:470. doi: 10.1097/QAI.0b013e31802f1318 PMC6276368 17179766

[9] Mills EJ Nachega JB Bangsberg DR Singh S Rachlis B Wu P . Adherence to HAART: a systematic review of developed and developing nation patient-reported barriers and facilitators. PloS Med. (2006) 3:e438. doi: 10.1371/journal.pmed.0030438 17121449 PMC1637123

[10] Mills JC Pence BW Todd JV Bengtson AM Breger TL Edmonds A . Cumulative burden of depression and all-cause mortality in women living with human immunodeficiency virus. Clin Infect Dis. (2018) 67:1575–81. doi: 10.1093/cid/ciy264 PMC6206117 29618020

[11] Sin NL DiMatteo MR . Depression treatment enhances adherence to antiretroviral therapy: a meta-analysis. Ann Behav Med. (2014) 47:259–69. doi: 10.1007/s12160-013-9559-6 PMC4021003 24234601

[12] Sheth SS Coleman J Cannon T Milio L Keller J Anderson J . Association between depression and nonadherence to antiretroviral therapy in pregnant women with perinatally acquired HIV. AIDS Care. (2015) 27:350–4. doi: 10.1080/09540121.2014.998610 25616659

[13] Desalegn SY Asaye MM Temesgan WZ Badi MB . Antenatal depression and associated factors among HIV-positive pregnant women in South Gondar zone public health facilities, northwest Ethiopia, a cross-sectional study. Clin Epidemiol Global Health. (2022) 16:101072. doi: 10.1016/j.cegh.2022.101072

[14] Nhiwatiwa S Patel V Acuda W . Predicting postnatal mental disorder with a screening questionnaire: a prospective cohort study from Zimbabwe. J Epidemiol Community Health. (1998) 52:262. doi: 10.1136/jech.52.4.262 9616415 PMC1756703

[15] Tuthill EL Pellowski JA Young SL Butler LM . Perinatal depression among HIV-infected women in KwaZulu-Natal South Africa: prenatal depression predicts lower rates of exclusive breastfeeding. AIDS Behav. (2017) 21:1691–8. doi: 10.1007/s10461-016-1557-9 PMC5393963 27752868

[16] Hartley M Tomlinson M Greco E Comulada WS Stewart J Le Roux I . Depressed mood in pregnancy: prevalence and correlates in two Cape Town peri-urban settlements. Reprod Health. (2011) 8:1–7. doi: 10.1186/1742-4755-8-9 21535876 PMC3113332

[17] Bonari L Pinto N Ahn E Einarson A Steiner M Koren G . Perinatal risks of untreated depression during pregnancy. Can J Psychiatry. (2004) 49:726–35. doi: 10.1177/070674370404901103 15633850

[18] Wagner GJ McBain RK Akena D Ngo V Nakigudde J Nakku J . Maternal depression treatment in HIV (M-DEPTH): study protocol for a cluster randomized controlled trial. Medicine. (2019) 98:27. doi: 10.1097/MD.0000000000016329 PMC6635242 31277180

[19] Dadi AF Miller ER Mwanri L . Postnatal depression and its association with adverse infant health outcomes in low-and middle-income countries: a systematic review and meta-analysis. BMC pregnancy childbirth. (2020) 20:1–15. doi: 10.1186/s12884-020-03092-7 PMC7374875 32698779

[20] Shen H Magnusson C Rai D Lundberg M Le-Scherban F Dalman C . Associations of parental depression with child school performance at age 16 years in Sweden. JAMA Psychiatry. (2016) 73:239–46. doi: 10.1001/jamapsychiatry.2015.2917 26842307

[21] Mathers CD Loncar D . Projections of global mortality and burden of disease from 2002 to 2030. PloS Med. (2006) 3:e442. doi: 10.1371/journal.pmed.0030442 17132052 PMC1664601

[22] Saxena S Funk M Chisholm D . World health assembly adopts comprehensive mental health action plan 2013–2020. Lancet. (2013) 381:1970–1. doi: 10.1016/S0140-6736(13)61139-3 23746771

[23] Health FDRoEMo . National Mental Health Strategy 2012/13–2015/16. Public policy document, Federal Democratic Republic of Ethiopia (2012).

[24] Abate HK Mekonnen CK . Depression among HIV-positive pregnant women at northwest Amhara referral hospitals during COVID-19 pandemic. Dove Press. (2021) 14:4897–905. doi: 10.2147/RMHP.S320311 PMC8665771 34908887

[25] Abebe W Gebremariam M Molla M Teferra S Wissow L Ruff A . Prevalence of depression among HIV-positive pregnant women and its association with adherence to antiretroviral therapy in Addis Ababa, Ethiopia. PloS One. (2022) 17:e0262638. doi: 10.1371/journal.pone.0262638 35051244 PMC8775187

[26] Harrington BJ Pence BW John M Melhado CG Phulusa J Mthiko B . Prevalence and factors associated with antenatal depressive symptoms among women enrolled in Option B+ antenatal HIV care in Malawi: a cross-sectional analysis. J Ment Health (Abingdon England). (2019) 28:198–205. doi: 10.1080/09638237.2018.1487542 PMC6440874 30270683

[27] Jones MU Esber AL Dear N Bahemana E Kibuuka H Iroezindu M . Ake JA et al: The pregnancy factor: the prevalence of depression among women living with HIV enrolled in the African Cohort Study (AFRICOS) by pregnancy status. Arch women’s Ment Health. (2021) 24:649–58. doi: 10.1007/s00737-021-01117-4 33683462

[28] Ngocho JS Watt MH Minja L Knettel BA Mmbaga BT Williams PP . Depression and anxiety among pregnant women living with HIV in Kilimanjaro region, Tanzania. PloS One. (2019) 14:e0224515. doi: 10.1371/journal.pone.0224515 31671160 PMC6822761

[29] Nyamukoho E Mangezi W Marimbe B Verhey R Chibanda D . Depression among HIV positive pregnant women in Zimbabwe: a primary health care based cross-sectional study. BMC pregnancy childbirth. (2019) 19:1–7. doi: 10.1186/s12884-019-2193-y 30704428 PMC6357405

[30] Osborn L Ronen K Larsen AM . Antenatal depressive symptoms in Kenyan women living with HIV: contributions of recent HIV diagnosis. stigma partner viol. (2022) 34:69–77. doi: 10.1080/09540121.2021.1981216 PMC8758509 34579601

[31] Peltzer K Rodriguez VJ Jones D . Prevalence of prenatal depression and associated factors among HIV-positive women in primary care in Mpumalanga province, South Africa. SAHARA-J: J Soc Aspects HIV/AIDS. (2016) 13:60–7. doi: 10.1080/17290376.2016.1189847 PMC4955403 27250738

[32] Regan M Muhihi A Saleh A Duggan CP Ulenga N Alwy Al-Beity FM . Antenatal depression and adverse birth outcomes among pregnant women living with HIV in Dar es Salaam, Tanzania. J Affect Disord. (2023) 339:82–8. doi: 10.1016/j.jad.2023.07.047 PMC10538406 37437720

[33] Shoptaw S Moucheraud C Mvududu R Essack Z Gorbach PM Myer L . Maliwichi M et al: Prevalence and incidence of probable perinatal depression among women enrolled in Option B+ antenatal HIV care in Malawi. J Acquir Immune Defic Syndr. (2018) 239:115–22. doi: 10.1016/j.jad.2018.06.001 PMC6089649 29990658

[34] Yator O Mathai M Vander Stoep A Rao D Kumar M . Risk factors for postpartum depression in women living with HIV attending prevention of mother-to-child transmission clinic at Kenyatta National Hospital, Nairobi. AIDS Care. (2016) 28:884–9. doi: 10.1080/09540121.2016.1160026 PMC4965230 27045273

[35] Habib NA Daltveit AK Bergsjø P Shao J Oneko O Lie RT . Maternal HIV status and pregnancy outcomes in northeastern Tanzania: a registry-based study. BJOG: Int J Obstet Gynaecol. (2008) 115:616–24. doi: 10.1111/j.1471-0528.2008.01672.x 18333943

[36] Lopez M Figueras F Hernandez S Lonca M Garcia R Palacio M . Association of HIV infection with spontaneous and iatrogenic preterm delivery: effect of HAART. Aids. (2012) 26:37–43. doi: 10.1097/QAD.0b013e32834db300 22008651

[37] Le Doare K Bland R Newell M-L . Neurodevelopment in children born to HIV-infected mothers by infection and treatment status. Pediatrics. (2012) 130:e1326–44. doi: 10.1542/peds.2012-0405 23118140

[38] Schwartz S Mudavanhu M Hanrahan C France H Nel J Mutunga L . Tibebu NS et al: Depression, anxiety and stress among HIV-positive pregnant women in Ethiopia during the COVID-19 pandemic. AIDS Care. (2023) 117:317–25. doi: 10.1093/trstmh/trac126 36579933

[39] Halim N Beard J Mesic A Patel A Henderson D Hibberd P . Intimate partner violence during pregnancy and perinatal mental disorders in low and lower middle income countries: A systematic review of literature, 1990–2017. Clin Psychol Rev. (2018) 66:117–35. doi: 10.1016/j.cpr.2017.11.004 29198412

[40] Leigh B Milgrom J . Risk factors for antenatal depression, postnatal depression and parenting stress. BMC Psychiatry. (2008) 8:1–11. doi: 10.1186/1471-244X-8-24 18412979 PMC2375874

[41] Page MJ McKenzie JE Bossuyt PM Boutron I Hoffmann TC Mulrow CD . The PRISMA 2020 statement: an updated guideline for reporting systematic reviews. Int J Surg. (2021) 88:105906. doi: 10.1016/j.ijsu.2021.105906 33789826

[42] Institute JB . JBI critical appraisal checklist for analytical cross sectional studies. Diakses pada. (2017) 22:2019–05.

[43] Borenstein M Hedges LV Higgins JP Rothstein HR . Introduction to meta-analysis. John Wiley & Sons (2021). doi: 10.1002/9781119558378

[44] Higgins JP Thompson SG . Quantifying heterogeneity in a meta-analysis. Stat Med. (2002) 21:1539–58. doi: 10.1002/sim.1186 12111919

[45] Cooper H Hedges LV Valentine JC . The handbook of research synthesis and meta-analysis. Russell Sage Foundation (2019). Available at: https://www.russellsage.org/publications/handbook-research-synthesis-and-meta-analysis-second-edition.

[46] Shidhaye P Giri P . Maternal depression: a hidden burden in developing countries. Ann Med Health Sci Res. (2014) 4:463–5. doi: 10.4103/2141-9248.139268 PMC4160664 25221688

[47] Bailey H Malyuta R Semenenko I Townsend CL Cortina-Borja M Thorne C . EuroCoord UECSi: Prevalence of depressive symptoms in pregnant and postnatal HIV-positive women in Ukraine: a cross-sectional survey. Reprod Health. (2016) 13:1–10. doi: 10.1186/s12978-016-0150-z 27000405 PMC4802605

[48] Zeng Y Cui Y Li J . Prevalence and predictors of antenatal depressive symptoms among Chinese women in their third trimester: a cross-sectional survey. BMC Psychiatry. (2015) 15:1–7. doi: 10.1186/s12888-015-0452-7 25879965 PMC4387591

[49] Bonacquisti A Geller PA Aaron E . Rates and predictors of prenatal depression in women living with and without HIV. AIDS Care. (2014) 26:100–6. doi: 10.1080/09540121.2013.802277 23750820

[50] Nasreen HE Kabir ZN Forsell Y Edhborg M . Prevalence and associated factors of depressive and anxiety symptoms during pregnancy: a population based study in rural Bangladesh. BMC women’s Health. (2011) 11:1–9. doi: 10.1186/1472-6874-11-22 21635722 PMC3117808

[51] Waldron EM Burnett-Zeigler I Wee V Ng YW Koenig LJ Pederson AB . Mental health in women living with HIV: the unique and unmet needs. J Int Assoc Providers AIDS Care (JIAPAC). (2021) 20:2325958220985665. doi: 10.1177/2325958220985665 PMC7829520 33472517

[52] Ettman CK Abdalla SM Cohen GH Sampson L Vivier PM Galea S . Prevalence of depression symptoms in US adults before and during the COVID-19 pandemic. JAMA netw Open. (2020) 3:e2019686. doi: 10.1001/jamanetworkopen.2020.19686 32876685 PMC7489837

[53] Stack RJ Meredith A . The impact of financial hardship on single parents: An exploration of the journey from social distress to seeking help. J Family econ Issues. (2018) 39:233–42. doi: 10.1007/s10834-017-9551-6 PMC5932102 29755247

[54] Baranowska-Rataj A Matysiak A Mynarska M . Does lone motherhood decrease women’s happiness? Evidence from qualitative and quantitative research. J Happiness Stud. (2014) 15:1457–77. doi: 10.1007/s10902-013-9486-z

[55] Biaggi A Conroy S Pawlby S Pariante CM . Identifying the women at risk of antenatal anxiety and depression: A systematic review. J Affect Disord. (2016) 191:62–77. doi: 10.1016/j.jad.2015.11.014 26650969 PMC4879174

[56] Yanikkerem E Ay S Mutlu S Goker A . Antenatal depression: prevalence and risk factors in a hospital based Turkish sample. J Pak Med Assoc. (2013) 63:472–7. 23905444

[57] Haider II . Antenatal depression and its predictors in Lahore, Pakistan. (2013). 23882957

[58] Rao D Feldman BJ Fredericksen RJ Crane PK Simoni JM Kitahata MM . A structural equation model of HIV-related stigma, depressive symptoms, and medication adherence. AIDS Behav. (2012) 16:711–6. doi: 10.1007/s10461-011-9915-0 PMC3314086 21380495

[59] Van Parys A-S Verhamme A Temmerman M Verstraelen H . Intimate partner violence and pregnancy: a systematic review of interventions. PloS One. (2014) 9:e85084. doi: 10.1371/journal.pone.0085084 24482679 PMC3901658

[60] Belay S Astatkie A Emmelin M Hinderaker SG . Intimate partner violence and maternal depression during pregnancy: a community-based cross-sectional study in Ethiopia. PloS One. (2019) 14:e0220003. doi: 10.1371/journal.pone.0220003 31365566 PMC6668805

