# Supplementary material for: Antenatal depression and its predictors among HIV positive women in Sub-Saharan Africa; a systematic review and meta-analysis
Source: Front Psychiatry. 2024 Jun 11;15:1385323. doi: 10.3389/fpsyt.2024.1385323 (PMC11196764; doi:10.3389/fpsyt.2024.1385323)
Supplement: Supplementary file 1 [file Table_1.docx]

Additional file 1: Search terms summary

| **Database** |  | **Query** | **Items found** |
| --- | --- | --- | --- |
| PubMed | **#1** | ((((((antenatal) OR (prenatal)) OR (antepartum)) OR (during pregnancy) | 54,001 |
|  | **#2** | (Depression) OR (Mental disorder) | 192,669 |
|  | **#3** | HIV | 42,139 |
|  | **#4** | **(((((((((((((((((((((((((((((((((((((((((((((((Angola) OR (Benin)) OR (Botswana)) OR (Burkina Faso)) OR (Burundi)) OR (Cameroon)) OR (Cape Verde)) OR (Central African)) OR (Chad)) OR (Comoros)) OR (Democratic Republic of Congo)) OR (Congo)) OR (Côte d'Ivoire)) OR (Djibouti)) OR (Eritrea)) OR (Eswatini)) OR (Ethiopia)) OR (Gabon)) OR (Gambia)) OR (Ghana)) OR (Guinea)) OR (Guinea Equatorial)) OR (Guinea-Bissau)) OR (Kenya)) OR (Lesotho)) OR (Liberia)) OR (Madagascar)) OR (Malawi)) OR (Mali)) OR (Mauritania)) OR (Mauritius)) OR (Mozambique)) OR (Namibia)) OR (Niger)) OR (Nigeria)) OR (Rwanda)) OR (Senegal)) OR (Seychelles)) OR (Sierra Leone)) OR (Somalia)) OR (South Africa)) OR (South Sudan)) OR (Sudan)) OR (Tanzania)) OR (Togo)) OR (Uganda)) OR (Zambia)) OR (Zimbabwe)** | 71,106 |
|  | **#5** | **#1 AND #2 AND #3 AND #4** | **485** |
| HINARI |  | ((antenatal) OR (prenatal) OR (antepartum) OR (perinatal) OR (during pregnancy) AND (Depression) OR (Mental disorder)) AND HIV | **1,846** |
| Web Of Science |  | ((antenatal) OR (prenatal) OR (antepartum) OR (perinatal) OR (during pregnancy) AND (Depression) OR (Mental disorder)) AND HIV AND Sub-Saharan African countries | **1,078** |
| Psychiatry Online |  | ((antenatal) OR (prenatal) OR (antepartum) AND (Depression) OR (Mental disorder)) AND HIV | **368** |
| PsycINFO |  | ((antenatal) OR (prenatal) OR (antepartum) AND (Depression) OR (Mental disorder)) AND HIV | **423** |
| Science Direct |  | ((antenatal) OR (prenatal) OR (antepartum) AND (Depression) OR (Mental disorder)) AND HIV | **3,076** |
| Google Scholar |  | ((antenatal) OR (prenatal) OR (antepartum) AND (Depression) OR (Mental disorder)) AND HIV | **98** |
| **Total** | | | **7,374** |
