# Supplementary material for: Antenatal depression and its predictors among HIV positive women in Sub-Saharan Africa; a systematic review and meta-analysis
Source: Front Psychiatry. 2024 Jun 11;15:1385323. doi: 10.3389/fpsyt.2024.1385323 (PMC11196764; doi:10.3389/fpsyt.2024.1385323)
Supplement: Supplementary file 2 [file Table_2.docx]

S1 Table: critical appraisal of studies included in the systematic review and meta-analysis for pooled prevalence of perinatal depression among HIV-positive women in Sub-Saharan Africa, 2024

| Included studies | Eight JBI Critical Appraisal Checklist for cross-sectional studies: The tool has Yes, No, Unclear, and Not Applicable options: “1” is given for “Yes” and “0” is given for other options | | | | | | | | |
| --- | --- | --- | --- | --- | --- | --- | --- | --- | --- |
|  | The criteria for inclusion in the sample clearly defined | Describe study setting and participant | Valid and reliable exposure measurement | Objective and standard criteria for measurement | Identified confounder | Strategies to deal with confounders | Valid and reliable outcome measurement | Appropriate statically analysis | Overall |
| Abate H. et. al., 2021 [[24](#_ENREF_24)] | 1 | 1 | 1 | 1 | 1 | 1 | 1 | 1 | 8/8= 100 |
| Abebe W. et. al., 2022 [[25](#_ENREF_25)] | 1 | 1 | 1 | 1 | 1 | 1 | 1 | 1 | 8/8= 100 |
| Harrington, B. et. al., 2019 [[26](#_ENREF_26)] | 1 | 1 | 1 | 1 | 1 | 1 | 1 | 1 | 8/8= 100 |
| Yator O. et. al., 2016 [[34](#_ENREF_34)] | 1 | 1 | 1 | 1 | 1 | 1 | 1 | 1 | 8/8=100 |
| Jones M. et. al., 2021[[27](#_ENREF_27)] | 1 | 1 | 1 | 1 | 1 | 1 | 1 | 1 | 8/8=100 |
| Osborn, L. et. al., 2022 [[30](#_ENREF_30)] | 1 | 1 | 1 | 1 | 1 | 1 | 1 | 1 | 8/8=100 |
| Regan, M. et. al., 2023[[32](#_ENREF_32)] | 1 | 1 | 1 | 1 | 1 | 0 | 1 | 1 | 7/8=87.5 |
| Schwartz, S. et. al., 2023[[35](#_ENREF_35)] | 1 | 1 | 1 | 1 | 1 | 1 | 1 | 1 | 8/8=100 |
| Shoptaw, S. et. al., 2018[[33](#_ENREF_33)] | 1 | 1 | 1 | 1 | 1 | 1 | 1 | 1 | 8/8=100 |
| Desalegn et. al., 2022 [[13](#_ENREF_13)] | 1 | 1 | 1 | 1 | 1 | 1 | 1 | 1 | 8/8=100 |
| Nyamukoho et. al., 2019 [[29](#_ENREF_29)] | 1 | 1 | 1 | 1 | 1 | 0 | 1 | 1 | 7/8=87.5 |
| Peltzer, et. al., 2016 [[31](#_ENREF_31)] | 1 | 1 | 1 | 1 | 1 | 1 | 1 | 1 | 8/8=100 |
| Ngocho, et. al., 2019 [[28](#_ENREF_28)] | 1 | 1 | 1 | 1 | 1 | 1 | 1 | 1 | 8/8=100 |
